# Core Outcome Measures for Trials in People With Coronavirus Disease 2019: Respiratory Failure, Multiorgan Failure, Shortness of Breath, and Recovery

**DOI:** 10.1097/CCM.0000000000004817

**Published:** 2021-01-05

**Authors:** Allison Tong, Amanda Baumgart, Nicole Evangelidis, Andrea K. Viecelli, Simon A. Carter, Luciano Cesar Azevedo, Tess Cooper, Andrew Bersten, Lilia Cervantes, Derek P. Chew, Sally Crowe, Ivor S. Douglas, Ella Flemyng, Julian H. Elliott, Elyssa Hannan, Peter Horby, Martin Howell, Angela Ju, Jaehee Lee, Eduardo Lorca, Deena Lynch, Karine E. Manera, John C. Marshall, Andrea Matus Gonzalez, Anne McKenzie, Sangeeta Mehta, Mervyn Mer, Andrew Conway Morris, Dale M. Needham, Saad Nseir, Pedro Povoa, Mark Reid, Yasser Sakr, Ning Shen, Alan R. Smyth, A. John Simpson, Tom Snelling, Giovanni F. M. Strippoli, Armando Teixeira-Pinto, Antoni Torres, Tari Turner, Steve Webb, Paula R. Williamson, Laila Woc-Colburn, Junhua Zhang, Jonathan C. Craig

**Affiliations:** 1 Sydney School of Public Health, The University of Sydney, Sydney, NSW, Australia.; 2 Centre for Kidney Research, The Children’s Hospital at Westmead, Sydney, NSW, Australia.; 3 Department of Nephrology, Princess Alexandra Hospital, Brisbane, QLD, Australia.; 4 Department of Critical Care Medicine, Hospital Sirio-Libanes, São Paulo, Brazil.; 5 College of Medicine and Public Health, Flinders University, Adelaide, SA, Australia.; 6 Department of Medicine, Denver Health, Denver, CO.; 7 Crowe Associates, Oxon, United Kingdom.; 8 Department of Medicine, Pulmonary Sciences and Critical Care, Denver Health, Denver, CO.; 9 School of Medicine, University of Colorado Anschutz, Denver, CO.; 10 Editorial and Methods Department, Cochrane, London, United Kingdom.; 11 Cochrane Australia, School of Public Health and Preventive Medicine, Monash University, Melbourne, VIC, Australia.; 12 Nuffield Department of Medicine, University of Oxford, Oxford, United Kingdom.; 13 Department of Internal Medicine, School of Medicine, Kyungpook National University, Daegu, South Korea.; 14 Department of Internal Medicine, Faculty of Medicine, University of Chile, Santiago, Chile.; 15 Department of Surgery, University of Toronto, Toronto, Canada.; 16 Department of Critical Care Medicine, University of Toronto, Toronto, Canada.; 17 Telethon Kids Institute, Perth, WA, Australia.; 18 Department of Medicine, Interdepartmental Division of Critical Care Medicine, University of Toronto, Toronto, ON, Canada.; 19 Division of Critical Care and Pulmonology, Department of Medicine, Charlotte Maxeke Johannesburg Academic Hospital, Johannesburg, South Africa.; 20 Faculty of Health Sciences, University of the Witwatersrand, Johannesburg, South Africa.; 21 Department of Medicine, University of Cambridge, Cambridge, United Kingdom.; 22 School of Medicine, Johns Hopkins University, Baltimore, MD.; 23 Critical Care Centre, CHU Lille, Lille University, Lille, France.; 24 Nova Medical School, CHRC, New University of Lisbon, Polyvalent Intensive Care Unit, Sao Francisco Xavier Hospital, CHLO, Lisbon, Portugal.; 25 Center for Clinical Epidemiology and Research Unit of Clinical Epidemiology, Odense University Hospital, Odense, Denmark.; 26 Department of Anesthesiology and Intensive Care, Jena University Hospital, Jena, Germany.; 27 Department of Respiratory Medicine, Peking University Third Hospital, Beijing, China.; 28 Evidence Based Child Health Group, University of Nottingham, Nottingham, United Kingdom.; 29 Translational and Clinical Research Institute, Newcastle University, Newcastle upon Tyne, United Kingdom.; 30 Sydney Children’s Hospital Network, Westmead, NSW, Australia.; 31 Wesfarmers Centre of Vaccines and Infectious Diseases, Telethon Kids Institute, Nedlands, WA, Australia.; 32 Department of Emergency and Organ Transplantation, University of Bari, Bari, Italy.; 33 Department of Pulmonology Hospital Clinic, University of Barcelona, CIBERESUCICOVID, IDIBAPS, ICREA, Barcelona, Spain.; 34 Department of Biostatistics, University of Liverpool, Liverpool, United Kingdom.; 35 Section of Infectious Diseases, Department of Medicine, National School of Tropical Medicine, Baylor College of Medicine, Houston, TX.; 36 Evidence-Based Medicine Center, Tianjin University of Traditional Chinese Medicine, Tianjin, China.

**Keywords:** clinical trial, coronavirus, critical care, infection, patients, sepsis

## Abstract

**OBJECTIVES:**

Respiratory failure, multiple organ failure, shortness of breath, recovery, and mortality have been identified as critically important core outcomes by more than 9300 patients, health professionals, and the public from 111 countries in the global coronavirus disease 2019 core outcome set initiative. The aim of this project was to establish the core outcome measures for these domains for trials in coronavirus disease 2019.

**DESIGN:**

Three online consensus workshops were convened to establish outcome measures for the four core domains of respiratory failure, multiple organ failure, shortness of breath, and recovery.

**SETTING:**

International.

**PATIENTS:**

About 130 participants (patients, public, and health professionals) from 17 countries attended the three workshops.

**INTERVENTIONS:**

None.

**MEASUREMENTS AND MAIN RESULTS:**

Respiratory failure, assessed by the need for respiratory support based on the World Health Organization Clinical Progression Scale, was considered pragmatic, objective, and with broad applicability to various clinical scenarios. The Sequential Organ Failure Assessment was recommended for multiple organ failure, because it was routinely used in trials and clinical care, well validated, and feasible. The Modified Medical Research Council measure for shortness of breath, with minor adaptations (recall period of 24 hr to capture daily fluctuations and inclusion of activities to ensure relevance and to capture the extreme severity of shortness of breath in people with coronavirus disease 2019), was regarded as fit for purpose for this indication. The recovery measure was developed de novo and defined as the absence of symptoms, resumption of usual daily activities, and return to the previous state of health prior to the illness, using a 5-point Likert scale, and was endorsed.

**CONCLUSIONS:**

The coronavirus disease 2019 core outcome set recommended core outcome measures have content validity and are considered the most feasible and acceptable among existing measures. Implementation of the core outcome measures in trials in coronavirus disease 2019 will ensure consistency and relevance of the evidence to inform decision-making and care of patients with coronavirus disease 2019.

## Abstract 2

Supplemental Digital Content is available in the text.

In March 2020, the World Health Organization declared coronavirus disease 2019 (COVID-19) a pandemic. COVID-19 is an acute respiratory illness that is life-threatening, largely because of respiratory failure (1–3). Severe and debilitating symptoms, including shortness of breath (dyspnea), are also very common in people with COVID-19 (18–71%) and can often be prolonged (4–12). Patients who have recovered from COVID-19 may still be at risk of long-term complications including lung fibrosis (13) and report debilitating ongoing symptoms such as dyspnea and fatigue, impaired quality of life, and a lingering inability to return to their usual activities (5, 8, 14–16). Patients have reported recovery periods lasting for more than 2 months (17).

In response to the pandemic, there has been a proliferation of trials. However, the wide variability of outcomes included and frequent omission of patient-reported outcomes including quality of life and symptoms can limit the usefulness of trials for decision-making (18–20). The global COVID-19 Core Outcomes Set (COVID-19-COS) initiative was launched in March 2020 (17, 21–23). More than 9300 patients, family members, members of the general public, and health professionals from over 110 countries participated in an international survey and consensus workshops to establish core outcome domains to be reported in trials in people with confirmed or suspected COVID-19, regardless of severity of disease (17, 22). Respiratory failure, multiple organ failure, shortness of breath, recovery, and mortality were identified as core outcome domains (17, 22). Despite being highly prioritized, these outcomes remain infrequently and inconsistently measured across trials in COVID-19, which limits the relevance and ability to compare the effect of interventions across trials.

Developing a standardized set of core outcome measures that are relevant and meaningful to people with COVID-19 and are feasible to implement in all trials can strengthen the evidence base for decision-making and can reduce research waste. Other outcomes may be included in trials for different patient populations (e.g., based on severity of disease) and care settings (e.g., intensive care, outpatient, and community). As part of the COVID-19-COS initiative, we convened a series of international online consensus workshops to establish core outcome measures for trials in people with suspected or confirmed COVID-19. Of note, a core outcome measure already exists for mortality (20). This report summarizes the workshop discussions on establishing a core outcome measure for respiratory failure, multiple organ failure, shortness of breath, and recovery, and presents the recommended measures to be used in trials in people with COVID-19.

## MATERIALS AND METHODS

### Overview and Context

Three online workshops were convened to discuss a core outcome measure for respiratory failure and multiple organ failure (combined workshop), shortness of breath, and recovery. These measures were intended to be for trials in people with confirmed or suspected COVID-19 across the spectrum of severity of disease and care settings (from community to hospital, and low- to middle-income countries to high-income countries), irrespective of the intervention. The workshops were convened using Zoom videoconferencing from May 13, 2020, to May 19, 2020. We used the core outcome measures in effectiveness (COMET) framework to inform this process (24, 25). In the context of the pandemic and rapid proliferation of trials of interventions for people with COVID-19, our explicit focus was to identify the best available existing measures and to ensure content validity and feasibility in the first instance and to develop a new measure only if required.

### Participants and Contributors

People with confirmed or suspected COVID-19 aged 18 years old and older, family members, members of the general public, and health professionals (including multidisciplinary clinicians, researchers, funders, policy makers, and stakeholders from research and trial organizations including ClinicalTrials.gov) were invited by members of the COVID-19-COS Steering Committee and Investigators using standardized invitation emails. Patients were also identified through Facebook and Twitter posts. In total, 130 participants (including 25 people with suspected or confirmed COVID-19 and members of the public) from 17 countries, including Australia, Belize, Brazil, Canada, Chile, China Mainland and special administrative regions, Ecuador, France, Germany, Italy, Portugal, South Africa, Spain, The Netherlands, United Kingdom, Uruguay, and the United States, attended the three workshops. The list of attendees at each workshop and workshop investigators is provided in the Acknowledgments. Investigators who were unable to attend the workshop provided feedback on the workshop program and draft report.

### Workshop Program and Process

During each workshop, we presented the COVID-19-COS process and core outcome domains (17, 22), definitions (for respiratory failure [26], multiple organ failure [27–29], shortness of breath [30, 31], and recovery [17, 22, 32]), an overview of the measurement properties and feasibility considerations (24, 33), and a summary of existing measures based on a review of existing core outcome measures (20, 21, 27, 28, 34–38), registered and published trials in COVID-19 (19), and reviews of trials and measures in respiratory disease and critical illness (39–44). Existing measures were assessed using the COMET initiative and COnsensus-based Standards for the selection of health Measurement INstruments (COSMIN) framework (24, 33) to identify a proposed core outcome measure for discussion. An outline of each of the workshop presentations is provided in **Supplemental Digital Content 1–3** (http://links.lww.com/CCM/G115). A summary of the proposed measures for each core outcome domain is provided in the following section.

#### Respiratory Failure.

The proposed outcome measure for respiratory failure was based on the type and level of respiratory support as captured in the WHO Clinical Progression Scale. The original WHO Clinical Progression Scale is an 11-point scale (scores 0–10), of which scores 4–9 contain a measure of respiratory failure: no oxygen support required (~WHO score 4), oxygen by mask or nasal prongs (~WHO score 5), oxygen by noninvasive ventilation or high flow (~WHO score 6), intubation and mechanical ventilation, Po_2_/Fio_2_ mm Hg ≥ 150 or Spo_2_/Fio_2_ mm Hg ≥ 200 (~WHO score 7), intubation and mechanical ventilation, Po_2_/Fio_2_ mm Hg < 150 or Spo_2_/Fio_2_ mm Hg < 200 (~WHO score 8), and extracorporeal membrane oxygenation (ECMO) (included as option in WHO score 9) (20).

#### Multiple Organ Failure.

The WHO Clinical Progression Scale includes mechanical ventilation and vasopressors, dialysis, or ECMO (22). Other common measures frequently used in trials in COVID-19 included the Sequential Organ Failure Assessment (SOFA) or quick SOFA (systolic blood pressure, respiratory rate, and Glasgow coma scale) and the Multiple Organ Dysfunction Score (MODS).

#### Shortness of Breath.

The Modified Medical Research Council (MMRC) Dyspnea Scale (45) was the best available measure based on COMET/COSMIN framework. The MMRC is well-validated and has been used to assess dyspnea in trials across different respiratory conditions, including COVID-19, and is simple to administer and generally more feasible to implement compared with other measures presented for comparison, including the modified Borg and Visual Analog Scale (VAS). The modified Borg did not capture exertion explicitly (and was not anchored to specific activities). The VAS was more abstract, was subjective, did not capture exertion explicitly, and was not feasible to implement. The modified Borg and VAS did not assess shortness of breath in terms of resumption of activities. The potential advantages and limitations for the three measures are provided in Supplemental Digital Content 1 (http://links.lww.com/CCM/G115).

#### Recovery.

The consensus-based definition of recovery as determined by patients and clinicians included the absence of symptoms related to the illness, the ability to do usual daily activities, and a return to a previous state of health and mind (prior to the COVID-19 illness) (17). No existing measures were found to provide an overall assessment of recovery, which captured all three dimensions of recovery important to patients with COVID-10 (17); thus, we developed and piloted a new COVID-19-COS recovery measure that was proposed to workshop participants. We adhered to the COMET/COSMIN framework as was feasible. The proposed COVID-19-COS recovery measure (Supplemental Digital Content 3, http://links.lww.com/CCM/G115) was piloted with nine patients and family members who confirmed that the measure was relevant to their experience of recovery and was comprehensible, and that it was easy to judge their level of recovery and generate a response.

Participants were allocated to virtual mixed breakout groups of eight to 10 attendees to ensure diverse discussion. A facilitator moderated each group using the discussion questions shown in Supplemental Digital Content 1–3 (http://links.lww.com/CCM/G115). The groups reconvened to provide a brief summary of their discussions.

All of the discussions were audiorecorded and transcribed. The transcripts were imported into HyperRESEARCH software. Authors (A.Tong, A.B., A.J., A.K.V.) coded the transcripts to identify themes and recommendations. These were reviewed and discussed among the facilitator team. All attending and nonattending investigators were invited to provide feedback on the draft report, and comments were integrated into the final version.

## RESULTS

The summary of the workshop themes and recommendations, and the COVID-19-COS core outcome measures for respiratory failure, multiple organ failure, shortness of breath, and recovery are provided below. Detailed explanations of the themes and supporting quotations are provided in Supplemental Digital Content 1–3 (http://links.lww.com/CCM/G115).

### Respiratory Failure

#### Summary of Themes.

The advantages of the WHO Progression scale were captured in three themes: simple and pragmatic, objective, consistent, and broad applicability. The limitations were lack of validation and granularity (e.g., it did not separate respiratory failure from other organ dysfunction), indirectness, and context dependence.

#### Core Outcome Measure for Respiratory Failure

The WHO core outcome measure of clinical progression could be used to assess respiratory failure based on the need for respiratory support (scores 4–9), with some minor adaptions (**Table 1**). We removed vasopressors and dialysis (in scores 8 and 9) to separate respiratory failure from other types of organ dysfunction and failure. Adding an oxygen saturation cutoff was suggested for circumstances where respiratory support was not available to capture the degree of hypoxemia; however, it was noted that measuring oxygen saturation in these circumstances may also not be available.

**TABLE 1.** Core Outcome Measure for Respiratory Failure

Based on minor adaptations to the WHO Clinical Progression Scale (22), the need for respiratory support is defined by the following:
No oxygen therapy (WHO score 4)
Oxygen by mask or nasal prongs (WHO score 5)
Oxygen by noninvasive ventilation or high flow (WHO score 6)
Intubation and mechanical ventilation, Po_2_/Fio_2_ ≥ 150 or Spo_2_/Fio_2_ ≥ 200 (WHO score 7)
Mechanical ventilation, Po_2_/Fio_2_ < 150 or Spo_2_/Fio < 200 (with the exclusion of vasopressors) (WHO score 8)
Extracorporeal membrane oxygenation (with the exclusion of other organ support options of vasopressor/dialysis) (WHO score 9)

### Multiple Organ Failure

#### Summary of Themes.

SOFA was preferred as the core outcome measure over the MODS. The advantages of the SOFA score were that it is was routinely used in research and practice, well validated particularly in ICU settings, and enabled ease of data collection. The limitations related to measurement errors related to clinical outcomes (death as a competing event and underlying comorbidities could bias the SOFA score), inadequate assessment of coagulopathy in the setting of COVID-19, limited use, and validation in non-ICU settings.

#### Core Outcome Measure

The SOFA score is recommended as a core outcome measure for multiple organ failure (**Table 2**).

**TABLE 2.** Core Outcome Measure for Multiple Organ Failure

0	1	2	3	4
Neurologic system (Glasgow Coma Scale)				
15	13–14	10–12	6–9	< 6
Respiratory System (Pao_2_/Fio_2_)				
> 400	301–400	≤ 30	101–200 + ventilation	≤ 100 + ventilation
Cardiovascular system (blood pressure/pressure-adjusted heart rate)				
No hypotension	MAP < 70 mmHg	Vasopressors^a^	Vasopressors^b^	Vasopressors^c^
Coagulation system (platelets × 10^3^/mm^3^)				
> 150	101–150	51–100	21–50	≤ 20
Hepatic system (bilirubin mcmol/L)				
< 20	20–32	33–101	102–204	204
Kidney system (creatinine mcmol/L, urine output)				
110	110–170	171–299	300–440 or 200–500 mL/d	> 440 or < 200 mL/d

^a^Dopamine ≤ 5 µg/kg/min or any amount of dobutamine.

^b^Dopamine > 5 µg/kg/min, epinephrine ≤ 0.1 mcg/kg/min or norepinephrine > 0.1 mcg/kg/min.

^c^Dopamine > 15 µg/kg/min or epinephrine > 0.1 mcg/kg/min.

The Sequential Organ Failure Assessment score adapted from Vincent et al (46).

### Shortness of Breath

#### Summary of Themes.

We identified three themes. Capturing the dynamic nature of COVID-19 meant recognizing the remitting trajectory of shortness of breath, reflecting the debilitating severity, and defining improvement in terms of resumption of normal activities. Comprehending the full experience of shortness of breath involved gaining awareness of an unfamiliar symptom, acknowledging the different sensations, and addressing the impact on mental health. Ensuring ease of implementation meant minimizing burden on patients with a simple and short measure, allowing flexibility in time points based on the population and intervention, and requiring validation.

#### Core Outcome Measure for Shortness of Breath

We made minor adaptations to the MMRC Dyspnea Scale (45) to ensure content validity and relevance to all phases of COVID-19 (**Table 3**). These are explained as follows:

**TABLE 3.** Core Outcome Measure for Shortness of Breath

Grade of Dyspnea	Description
0	I only got breathless with strenuous exercise (e.g., running and walking up a steep hill)
1	I got short of breath when hurrying on level ground or walking up a slight hill
2	On level ground, I walked slower than usual because of breathlessness or I had to stop for breath when walking at my own pace on the level
3	I stopped for breath after walking about 100 m or after a few minutes on level ground
4	I was breathless when dressing, talking or at rest

The measure is adapted from the modified Medical Research Council Dyspnea scale (45).

The instructions are shortness of breath refers to a diverse range of sensations associated with, but not limited to: feelings of tightness in the chest, not having enough air, being smothered, the need to breathe faster, heightened awareness of breathing, and being “hungry” for air. Please indicate the grade of shortness of breath, which best describes you in the last 24 hr.

1) An introduction, which specifies a range of sensations to describe shortness of breath, was provided, because this symptom may be new and unfamiliar to some patients with COVID-19.
2) The recall period was specified to be 24 hours due to daily fluctuations in the severity of shortness of breath.
3) Examples including running and walking up a steep hill were added to grade 0.
4) In grade 2, “people my age” was changed to “usual” to allow patients to compare their pace at the time of completion to what is considered normal for them, prior to the COVID-19 diagnosis, and to ensure relevance to children. This also reduced ambiguity as patients were not able to judge what the “normal” pace was for each age group. This also removed confusion about how to assess the average pace for each age group. Patients with an underlying health condition or those who naturally have a slower pace of walking than others did not find this phrase “people my age” to be meaningful.
5) In grade 3, “yards” was changed to “meters” in accordance with contemporary universal metrics.
6) In grade 4, the statement “to leave the house” was removed due to the need for self-quarantine in the context of COVID-19. The activities listed in grade 4 were modified (to include talking or at rest) to capture the extreme severity of shortness of breath in people with COVID-19.

### Recovery

#### Summary of Themes.

Three themes were identified. Recognizing the diverse meaning of recovery reflected the diversity of patients’ experiences that broadly included a return to the preillness state, complete resolution of symptoms, and physical and mental well-being. Addressing dynamic trajectories of recovery highlighted that recovery was not a linear process but followed a variable and unpredictable trajectory that needed to be distinguished from other comorbidities and the indirect effects of the pandemic. Ensuring feasible and universal implementation signaled a measure would need to be applicable to all care settings, populations, and severity of COVID-19, while having minimal burden on trial lists and patients to be implemented in all trials.

#### Core Outcome Measure for Recovery.

No existing measure captured the dimensions of recovery important to patients with COVID-19. The workshop recommendations regarding the measure were used to finalize the new core outcome measure for recovery (**Table 4**).

**TABLE 4.** Core Outcome Measure for Recovery

Not Recovered at All	Somewhat Recovered	About Half Recovered	Mostly Recovered	Completely Recovered
○	○	○	○	○


The instructions are “complete recovery” means you no longer have symptoms related to your illness and you can do your usual daily activities and you have returned to your previous state of health and mind (before your illness). Please choose the answer that best describes you today:

How recovered from your illness are you?

## DISCUSSION

Respiratory failure, multiple organ failure, shortness of breath, recovery, and mortality were identified as critically important core outcome domains by patients with COVID-19, their family members, the general public, and health professionals (17, 22). Here, we have identified the core outcome measures for all core outcome domains, except mortality, as the core measures have already been defined in the WHO Clinical Progression Scale (20). To facilitate implementation, the set of core outcome measures had to be meaningful and applicable to most if not all care settings, populations, and severity of COVID-19, while having minimal burden on trial lists and patients. Respiratory failure, measured by the need for respiratory support according the WHO Clinical Progression Scale (with minor modifications to distinguish respiratory failure from other types of organ dysfunction), was considered simple, practical, objective, and applicable to most clinical scenarios. The SOFA score as a core outcome measure for multiple organ failure was supported, because it was routinely used in clinical and research settings, well validated, and facilitated ease of data collection and reporting. The trajectory of the shortness of breath was unpredictable and fluctuated over time, could be extremely debilitating such that some were breathless when they were inactive, and impaired cognition and mental health. Some patients were initially unfamiliar with the sensation and could not recognize shortness of breath as a symptom, whereas others distinguished it from shortness of breath related to other conditions including asthma. Minor adaptations to the MMRC Dyspnea Scale were required to capture these patient-relevant dimensions. Recovery had to be interpreted within the individual patient contexts, and broadly, this included a return to normal activities, resolution of symptoms, and physical and mental well-being. Recovery followed a dynamic trajectory and needed to be distinguished from other comorbidities or indirect consequences of COVID-19 such as self-quarantine and unemployment. A new measure was developed to include the experience of recovery in patients with COVID-19.

Respiratory failure was already captured in the WHO minimal common outcome measure set for COVID-19 based on the need for respiratory support (20). However, it was acknowledged that assessing the degree of hypoxemia may require the addition of a measure of oxygen saturation. The SOFA score was the best available measure for multiple organ failure and had been validated in various populations including patients in ICU, and was a common and familiar measure. Both core outcome measures were generally simple to implement and parsimonious, as they were already established as a core measure (WHO clinical progression scale for respiratory failure) or routinely used in trials and clinical settings (SOFA for multiple organ failure).

Our findings suggest that some aspects of the experience of shortness of breath may be different from other conditions. The descriptors for asthma include incomplete exhalation and chest tightness, and for chronic obstructive pulmonary disease include increased work or effort to breath, suffocation, and air hunger (47, 48). For patients with COVID-19, shortness of breath was described as chest tightness or constriction. However, they emphasized that the severity fluctuated in an unpredictable manner, and at the most severe end of the spectrum, patients could be breathless even at rest. Minor changes were required to the MMRC Dyspnea Scale to ensure content validity, to reflect the experience of shortness of breath in patients with COVID-19, and for patients to comprehend and judge their grade of dyspnea using the response scale and descriptors. We explicitly added various examples of the sensation, so patients could recognize the symptoms, specified a 24-hr recall period to capture the fluctuations in severity. We also added descriptors to the last grade to capture fully the extreme severity of shortness of breath as experienced by patients with COVID-19.

Recovery from COVID-19 involves the resumption of normal activities and a return to the previous health state. Survivors of critical illness and patients with chronic obstructive pulmonary disease similarly evaluate their recovery from illness in terms of improving psychologic well-being, regaining a sense of control, and resuming their usual social roles and activities of daily living (49, 50). The COVID-19-COS recovery measure provides an overall assessment of recovery and captures recovery in way that is meaningful to patients and reflects their experience (content validity), is comprehensible to patients, and is easy and quick to complete. It distinguishes recovery after the COVID-19 illness from indirect social and economic effects of the pandemic and considers the impact of COVID-19 on the patient’s overall health state. Participants viewed the measure as applicable across all care settings and the full spectrum of the severity of COVID-19. A more detailed measure for recovery may be required for trials that assess recovery as a primary outcome.

There are limitations to the core outcome measures. Although a simple and succinct core outcome measure is necessary for broad implementation, some researchers may wish to assess separately the outcomes in detail using more granular measures to capture a more detailed understanding of the outcome. This may be required for trials in which respiratory failure, multiple organ failure, shortness of breath, or recovery are a primary outcome. Concurrent validation is needed for the core outcome measures, particularly for the patient-reported outcome measures for shortness of breath and recovery, to generate evidence to support other psychometric properties (such as construct validity, test-retest reliability, and responsiveness) in addition to content validity (33). Other measures may be more appropriate if a patient is unable to respond due to extenuating circumstances, such as young age, severe illness, or cognitive impairment. We recognize that it was not feasible to include in this process all countries, including those with large populations. We suggest that future efforts are needed to determine content validity for regions, including densely populated countries that were not included in this process.

## CONCLUSIONS

The COVID-19-COS set of core outcome measures was developed in partnership with patients, the public, and health professionals and are meaningful, feasible and simple, and broadly inclusive of most patient contexts and care settings. Consistent reporting of respiratory failure, multiple organ failure, shortness of breath, and recovery across trials in COVID-19 may enhance the relevance and value of the rapidly emerging evidence for decision-making and care of patients with COVID-19.

## ACKNOWLEDGMENTS

The authors thank with permission, all the patients, family members, members of the public, and health professionals who attended the consensus workshops:

Respiratory failure and multiple organ failure: Samaya Anumudi, Pedro Arriaga, Antonia Artigas, Dee Barlow, Jairo Barrantes Perez, Amanda Baumgart, Andrew Bersten, Simon Carter, Elaine Chan, Tess Cooper, Jonathan Craig, Fernando Cuellar, Keertan Dheda, Crispin Goytia, Jorge Hidalgo, Angela Ju, Jeanette Kusel, Karine Manera, Guy Marks, John Marshall, Mervyn Mer, Patricia Mesa, Andrew Conway Morris, Saad Nseir, Pedro Povoa, Gloria Rodriguez, Yasser Sakr, Carlos Sanchez, Manu Shankar-Hari, A John Simpson, Giovanni Strippoli, Armando Teixeira-Pinto, Allison Tong, Antoni Torres, Peter Tugwell, Andrea Viecelli, Paula Williamson, and Xinyue Zhang.

Shortness of breath: Samaya Anumudu, Pedro Arriaga, Jairo Barrantes Perez, Amanda Baumgart, Tess Cooper, Jonathan Craig, Fernando Cuellar, Claire DS, Nicole Evangelidis, Josh Fessel, Ella Flemyng, Rebecca Goodwin, Elyssa Hannan, Jorge Hidalgo, Angela Ju, Deena Lynch, Joanne Macdonald, Karine Manera, Guy Marks, Mervyn Mer, Patricia Mesa, Caroline Neely, Margaret O’Hara, Pedro Povoa, Gloria Rodriguez, Yasser Sakr, Carlos Sanchez, Manu Shankar-Hari, Ning Shen, A John Simpson, Robert Star, Giovanni Strippoli, Alix Surber, Armando Teixeira-Pinto, Allison Tong, Antoni Torres, Peter Tugwell, Ravichandran Veerasamy, Andrea Viecelli, David Williams, Paula Williamson, Tracy Workman, and Junhua Zhang.

Recovery: Jairo Barrantes-Perez, Amanda Baumgart, Simon Carter, Tess Cooper, Jonathan Craig, Sally Crowe, Christine De Leon, Nicole Evangelidis, Zachary Feiger, Josh Fessel, Ella Flymyng, Paul Garner, Rebecca Goodwin, Cristina Granja, Elyssa Hannan, Angela Ju, Alasdair Jubb, Lynn Laidlaw, Eduardo Lorca, Joanne Macdonald, Karine Manera, Andrea Matus Gonzalez, Charlie McLeod, Mervyn Mer, Patricia Mesa, Julian Morgans, Andrew Conway Morris, Dale Needham, Caroline Neely, Belinda Ng, Margaret O’Hara, Pedro Povoa, Otavio Ranzani, Karen Rawden, Sara Roloff, Alan Smyth, Robert Star, Giovanni Strippoli, Alix Surber, Armando Teixeira-Pinto, Caroline Terwee, Allison Tong, Antoni Torres, Sean Tunis, Andrea Viecelli, David Williams, Paula Williamson, Laila Woc-Colburn, and Tracy Workman.

The views expressed in the article do not represent the official position of any institutions of the collaborators.

Professor Simpson is a National Institute for Health Research (NIHR) Senior Investigator. The views expressed in this article are those of the author(s) and not necessarily those of the NIHR, or the Department of Health and Social Care.

## Supplementary Material



## APPENDIX 1. COVID-19 Core Outcomes Set Investigators (Attending and Nonattending Contributors) for Group Authorship

**Table:**

First Name	Last Name	Institution	Country
Aborode	Abdullah	Consumer—patient	Nigeria
Samaya	Anumudu	Baylor College of Medicine	United States
Pedro	Arriaga	Karl Heusner Memorial Hospital	Belize
Luciano	Azevedo	University of São Paulo	Brazil
Dee	Barlow	Consumer—patient	United Kingdom
Jairo	Barrantes Perez	Baylor College of Medicine	United States
Amanda	Baumgart	The University of Sydney	Australia
Andrew	Bersten	Flinders University	Australia
Fernando	Bozza	National Institute of Infectious Disease	Brazil
Simon	Carter	The University of Sydney	Australia
Lilia	Cervantes	University of Colorado	United States
Elaine	Chan	Westmead Hospital	Australia
Derek	Chew	Flinders University	Australia
Andrew	Conway Morris	University of Cambridge	United Kingdom
Tess	Cooper	The University of Sydney	Australia
Jonathan	Craig	Flinders University	Australia
Sally	Crowe	Crowe Associates Ltd	United Kingdom
Fernando	Cuellar	Karl Heusner Memorial Hospital	Belize
Christine	De Leon	Consumer—patient	United Kingdom
Keertan	Dheda	University of Cape Town	South Africa
Ivor	Douglas	Denver Hospital	United States
Claire	DS	Consumer—patient	United Kingdom
Julian	Elliott	Monash University	Australia
Nicole	Evangelidis	The University of Sydney	Australia
Ella	Flemyng	Cochrane	United Kingdom
Elyssa	Hannan	The University of Sydney	Australia
Paul	Garner	Liverpool School of Tropical Medicine	United Kingdom
Crispin	Goytia	Consumer—patient	United States
Cristina	Granja	University of Porto, Centro Hospitalar Universitario Sao Joao	Portugal
Adele	Haverlid	Consumer—patient	Sweden
Jorge	Hidalgo	Karl Heusner Memorial Hospital	Belize
Peter	Horby	University of Oxford	United Kingdom
Martin	Howell	The University of Sydney	Australia
Tamara MJ	Johnson	Consumer—patient	United States
Angela	Ju	The University of Sydney	Australia
Alasdair	Jubb	University of Cambridge	United Kingdom
Jeanette	Kusel	National Institutes for Clinical Excellence	United Kingdom
Lynn	Laidlaw	Consumer—public	United Kingdom
Steven	Landowski	Consumer—patient	United States
Jaehee	Lee	Kyungpook National University	South Korea
Eduardo	Lorca	University of Chile	Chile
Deena	Lynch	Consumer—patient	Australia
Joanne	Macdonald	University of the Sunshine Coast	Australia
Karine	Manera	The University of Sydney	Australia
Guy	Marks	University of New South Wales	Australia
John	Marshall	University of Toronto	Canada
Andrea	Matus Gonzalez	The University of Sydney	Australia
Anne	McKenzie	Telethon Kids Institute	Australia
Charlie	McLeod	Perth Children’s Hospital	Australia
Sangeeta	Mehta	University of Toronto	Canada
Mervyn	Mer	University of Witwatersrand Johannesburg	South Africa
Patricia	Mesa	Pasteur Hospital	Uruguay
Julian	Morgans	Consumer—patient	Australia
Dale	Needham	Johns Hopkins University	United States
Belinda	Ng	Consumer—patient	Hong Kong
Saad	Nseir	Lille University	France
Margaret	O’Hara	Consumer—patient	United Kingdom
Pedro	Povoa	Centro Hospitalar Lisboa Ocidental	Portugal
Otavio	Ranzani	Barcelona Institute for Global Health—ISGlobal	Spain
Karen	Rawden	Counselling and Psychotherapy	United Kingdom
Mark	Reid	Denver Hospital	United States
Gloria	Rodríguez-Vega	HIMA San Pablo-Caguas	Puerto Rico
Sara	Roloff	Consumer—patient	United Kingdom
Yasser	Sakr	University of Jena	Germany
Carlos	Sanchez	Hospital General IESS Quevedo	Ecuador
Manu	Shankar-Hari	Kings College London	United Kingdom
Ning	Shen	Peking University Third Hospital	China
A John	Simpson	Newcastle University, Newcastle Upon Tyne	United Kingdom
Alan	Smyth	University of Nottingham	United Kingdom
Tom	Snelling	The University of Sydney	Australia
Cheryl	Stevens	Consumer—patient	United Kingdom
Giovanni	Strippoli	University of Bari	Italy
Charlotte	Summers	University of Cambridge	United Kingdom
Alix	Surber	Consumer—patient	United States
Davide	Tarantino	Consumer-patient	Italy
Armando	Teixeira-Pinto	The University of Sydney	Australia
Caroline	Terwee	Amsterdam UMC	The Netherlands
Allison	Tong	The University of Sydney	Australia
Antoni	Torres	University of Barcelona	Spain
Peter	Tugwell	University of Ottawa	Canada
Sean	Tunis	FDA	United States
Tari	Turner	Monash University	Australia
Nina	Ustinoff	Consumer—patient	United Kingdom
Andrea	Viecelli	University of Queensland	Australia
Steve	Webb	Monash University	Australia
David	Williams	RTI	United States
Paula	Williamson	University of Liverpool	United Kingdom
Laila	Woc-Colburn	Baylor College of Medicine	United States
Tracy	Workman	Consumer - family member	United States
Junhua	Zhang	Tianjin University of Traditional Chinese Medicine	China
