# Supplementary material for: Core Outcome Measures for Trials in People With Coronavirus Disease 2019: Respiratory Failure, Multiorgan Failure, Shortness of Breath, and Recovery
Source: Crit Care Med. 2021 Jan 5;49(3):503–16. doi: 10.1097/CCM.0000000000004817 (PMC7892260; doi:10.1097/CCM.0000000000004817)
Supplement: Supplementary file 1 [file ccm-49-0503-s001.docx]

**Supplemental Digital Content 1: Respiratory failure and multiorgan failure: workshop presentation outline with review of measures**

NB. This was a combined workshop that included both respiratory failure and multiorgan failure

WORKSHOP PRESENTATION OUTLINE

**BRIEF OUTLINE OF RATIONALE**

Respiratory failure and multiorgan failure were identified as high priority outcome domains by patients, family members, health professionals and members of the general public through the international COVID-19-COS survey and consensus workshops.

**AIM OF THE WORKSHOP**

To decide on a core outcome measure for respiratory failure and for multiorgan failure for trials in people with confirmed or suspected COVID-19

**DEFINITIONS**

**Respiratory failure:** a condition in which not enough oxygen passes from your lungs into your blood. Your body's organs, such as your heart and brain, need oxygen-rich blood to work well. Respiratory failure also can occur if your lungs can't properly remove carbon dioxide (a waste gas) from your blood. Too much carbon dioxide in your blood can harm your body's organs. (NHLBI)

**Multiorgan failure:** refers to altered function in two or more organ systems in an acutely ill patient such that homeostasis cannot be maintained without intervention. (ACCP, SCC 1991)

**MEASUREMENT PROPERTIES AND FEASIBILITY**

| **Property** | **Explanation** |
| --- | --- |
| Validity | Whether the outcome measures assess the outcome (respiratory failure, multiorgan failure) it claims to assess accurately |
| Reliability | Whether the outcome can be measured in a reproducible way |
| Responsiveness | Whether the measure discriminates when change occurs |
| Feasibility | - Easy to measure? (expertise, equipment, time requirement) - Applicable across different settings? (hospital/ICU versus community) - Available in different settings? (low/middle/high income countries) |
| Acceptability | Easy to understand and interpret the results |

**EXISTING MEASURES FOR RESPIRATORY FAILURE**

**Core outcome sets**

- Meta-COS for COVID-19 (WHO, Jin et al. and Qiu et al.): Type of respiratory support (mask or nasal prongs, intubation and mechanical ventilation, ECMO)
- COVenT (ventilation trials): Time to extubation, time to reintubation, duration of mechanical ventilation

*Respiratory failure considered as part of a continuum (WHO Clinical Progression Scale 5 to 9)^22^

**Trials in COVID-19 (124/301 trials)**

- Need for respiratory support (mask or nasal prongs, intubation and mechanical ventilation, ECMO)
- Duration of mechanical ventilation (19%)
- Metrics: rate, number/proportion, duration

**Other endorsed/common measures**

- Berlin Definition of ARDS
- Murray score for acute lung injury
- Pulse oximetry (surrogate)
- Arterial blood gas test (surrogate)

**EXISTING MEASURES FOR MULTIORGAN FAILURE**

**Core Outcome Sets**

- WHO: Mechanical ventilation and vasopressors, dialysis or ECMO^22^

Consider other measures of organ dysfunction

- *Sequential Organ Failure Assessment (SOFA)*
- *Multiple Organ Dysfunction Score (MODS)*
- *Acute Coronary Syndrome*
- *Delirium*

**Trials in COVID-19 (25/301 trials)**

- Sequential Organ Failure Assessment (SOFA) (56% trials)
- Metrics: rate, number/proportion, duration, scores

**Other endorsed/common measures**

- qSOFA (systolic blood pressure, respiratory rate, GCS)

**Comparison between SOFA and MODS**

**SOFA=blue**

**MODS=orange**

| **0** | **1** | **2** | **3** | **4** |
| --- | --- | --- | --- | --- |
| **Neurological system (Glasgow Coma Scale)** | | | | |
| 15  15 | 13-14  13-14 | 10-12  10-12 | 6-9  7-9 | <6  ≤6 |
| **Respiratory system (PaO_2_/FiO_2_)** | | | | |
| >400  >300 | 301-400  226-300 | ≤30  151-225 | 101-200 + ventilation  76-150 | ≤100 + ventilation  ≤75 |
| **Cardiovascular system (blood pressure / pressure-adjusted heart rate)** | | | | |
| No hypotension  ≤10.0 | MAP <70mmHg  10.1-15.0 | Vasopressors^a^  15.1-20.0 | Vasopressors^b^  20.1-30.0 | Vasopressors^c^  >30 |
| **Coagulation system (plateletsx10^3^/mm^3^)** | | | | |
| >150  >120 | 101-150  81-120 | 51-100  51-80 | 21-50  21-50 | ≤20  ≤20 |
| **Hepatic system (bilirubin mcmol/L)** | | | | |
| <20  ≤20 | 20-32  21-60 | 33-101  61-120 | 102-204  121-240 | >204  >240 |
| **Kidney system (creatinine mcmol/L, urine output)** | | | | |
| 110  ≤20 | 110-170  101-200 | 171-299  201-350 | 300-440 or  200-500ml/day  351-500 | >440 or <200ml/day  >500 |

^a^dopamine ≤5 mcg/kg/min or any amount of dobutamine

^b^dopamine >5 mcg/kg/min, epinephrine ≤0.1mcg/kg/min or norepinephrine >0.1mcg/kg/min

^c^dopamine >15 mcg/kg/min or epinephrine >0.1mcg/kg/min

**DISCUSSION QUESTIONS**

**Respiratory failure**

1. Should it be defined as need for respiratory support (e.g. oxygen, mechanical ventilation, ECMO)? If so, would the WHO measure of clinical progression be suitable? Why/why not?
2. If interventions are not available, can ”need” be hypothetical i.e. based on other clinical assessment (symptoms, blood oxygen)?
3. Should respiratory failure be measured as duration (with/free of), proportion and/or rate?

**Multiorgan failure**

1. Should multiorgan failure be measured using the SOFA or MODS? Consider – suitability for trials in COVID-19; meaningfulness, feasibility and applicability, settings (ICU, non-ICU)

WORKSHOP THEMES AND QUOTATIONS

| **Themes** | **Quotations** |
| --- | --- |
| **Respiratory failure (need for respiratory support based on the WHO Clinical Progression Scale)** | |
| **Advantages** | |
| - **Simple and pragmatic:** The WHO scale was considered easy to use, with well-defined categories. The use of the WHO scale would avoid the need to introduce a new core outcome measure for respiratory failure, which could cause confusion. Also, interrelatedness amongst the items was an advantage from a methodology perspective (internal consistency). - **Objective:** The scale provided an objective measures and included hard clinical endpoints. - **Consistent and broad applicability:** The measure was applicable to most clinical scenarios and it was expected to be reported consistently across different sites. | “It's a good measure in terms of being a well-defined time blind and applying that you can actually define also in low resource settings. It is a good measure of, for the start of deterioration, but again you have to combine it with other measure that indicates respiratory failure.” – H1  “That's why most of the respiratory failure studies or the studies on ARDS looked at ventilator free days as a surrogate measure to measure the improvement of respiratory failure over time.” – H1  “If interventions are not available, it can need be hypothetical. I think so.” – H1  “This is the most sort of objective measure that we can get, it would be more or less consistently reported across different sites. There is the consideration that this measures the decision and decisions could differ in different places in terms of at what point you would mechanically ventilate the patient. In terms of objectivity, it's a good one.” – H1  “We should try and align with the WHO measures otherwise it's going to be very confusing for research if there's this WHO core outcome measure set and then there's an additional core outcomes set with respiratory support measured in a different way.” – H1  “One good thing about the WHO definition is the interrelatedness amongst the items from a methodology perspective with the internal consistency argument.” – H1  “The issue about need for respiratory support given being not entirely linked to respiratory failure, but rather somebody's assessment of what they need. That's less important of an issue in a randomised trial than it is in an observational study because the subjectivity would ideally be balanced across the two groups. I agree with the outcome definition being need for respiratory support.” – H1  “I like the elements of the WHO scale for hospitalised patients. It is important to use the appropriate definition for the appropriate setting.” – H1  “But in COVID, it's mainly oxygenation problem. Defining it in terms of oxygen, the success, or otherwise, of oxygenation is probably as good a way as any of measuring it.” – H1  “It allows almost all imaginable clinical scenarios. It's simple. It's pragmatic.” – H2  “It does make sense.” – P2  “The minute that you're needing supplemental, the oxygen kicks in that would automatically count as respiratory failure or some sort of respiratory support being needed. I'm okay with that definition.” – H2  “I do agree that the definition of the outcome based on the provision of the intervention is a critical. I agree with using the scale.” – H2  “It captures an endpoint and disease severity; this is its major advantage.” – H3 |
| **Limitations** |  |
| - **Lack of validation and granulatory:** Data to support the validity of the WHO progress scale were not available. The measure included respiratory failure and other organ dysfunction (e.g. cardiovascular, kidneys), which were not separated from respiratory failure. They also considered that it may not be adequately granular for patients defined as ambulatory (WHO Score 1-3). The scale measured evolution of the disease, not a specific outcome. - **Indirectness:** In assessing need for respiratory support, it did not capture respiratory failure in a direct manner. However, this may be less relevant in randomised trials. Some suggested that the measure may need to be combined with other measures of respiratory failure, depending on the trial. - **Context-dependence:** Participants recognised that the criteria for respiratory support (mechanical ventilation) varied among centres and countries. Clinical decision-making for managing respiratory failure was dynamic and variable, and could also depend on patient preferences. In some circumstances, patients may prefer not to receive mechanical ventilation. While the scale was dependent on resources and context, this may be less relevant in implementation in randomised trials. | “The indication for mechanical ventilation can vary from one institution to another. If you take it as a solid end point, you actually don't mark this respiratory support or respiratory failure, you mark actually a reaction to respiratory failure.” – H1  “The issue is around the validity of the instrument and the hypoxemic respiratory failure, the Berlin definition, provides a reasonable construct and it can be modified for resource limited settings using the Kigali modification of the Berlin definition for respiratory failure. If you were to use the Berlin definition for respiratory failure it also links up with the SOFA definition” – H1  “So the P/F ratio would not be actually the most appropriate way to do that. It's the more precise way, but it's not the most appropriate way.” – H1  “It's a scale actually to categorize that evolution of the disease. It combines actually a lot of parameters together like respiratory failure, for example, and vasopressor therapy. It's not a way to separate respiratory failure from other types of organ dysfunction and failure.” – H1  “Duration of respiratory failure very tricky because it depends on how you wean the patients or how the other organ failures evolve over time. And it can be subjected to a lot of individual and inter-centre variabilities. The duration of mechanical ventilation itself is not really a good measure to mirror the outcome of respiratory failure.” – H1  “The different criteria for mechanical ventilation will vary amongst countries institutions.” – H1  “The word mechanical ventilation and ventilators do ring a chilling bell in most patients and family members. They would actually tell you they would not want to be mechanically ventilated if ever the need arise. That's also something we need to think about, the consent for mechanical ventilation by patients or by family members.” – H1  “But I take the point that particularly on a score eight it's mechanical ventilation or vasopressors, which could mean that someone's on a vasopressor and that isn't a measure of respiratory support.” – H1  “Some sites don't have the resources, but you're adding much more subjectivity in when you're looking at symptoms, would be harder to make consistent across trials. When you talk about blood oxygen, we're talking about surrogate measures and for decision makers, we want hard clinical end points and to make decisions based on that, I would try to stay away from surrogate outcomes.” – H1  “The fundamental issues is not to link an organ failure with an organ support definition.” – H1  “The decision to provide an intervention is often independent or not entirely dependent on the clinical condition.” – H1 |
| **Suggestions for alternatives** |  |
| - Failure of improving oxygenation (PaO2 or O2 Sat) on maximum available respiratory support - Ventilator-free days - Percentage of patients requiring what type of respiratory support   - No support   - Nasal prong   - Facial mask   - Facial mask with reservoir   - High flow   - Non-invasive ventilation   - Invasive mechanical ventilation   - ECMO   - Respiratory support required but not available or applied - Duration of respiratory support or duration free of respiratory support - Berlin definition for ARDS (the most common type of hypoxic respiratory failure in COVID-19), using Kigali modification for resource limited settings^39^ | “Failure of improving PO2 on available or maximum respiratory support measures that you have in your institution.” – H1  “The need for mechanical ventilation as a solid endpoint and/or other physiological variables that indicate respiratory failure.” – H1  “Can we think about clinical and very basic points of measurements like respiration rate, oxygen saturation, chest x-ray. Things that are very available to everybody across the world, instead of the need for ECMO, for example, which isn't available in a lot of different places. And we would define the measure, the outcome based upon the actual objective clinical that's really simple to measure.” – H1  “We should measure duration and there is enough data out there for both either duration free of organ support or duration of organ support and both set at a particular time point, something like 28 days and 30 days have been proposed in ARDS literature.” – H1  “You do need to have some measurements of hypoxemia in order to define ventilatory or respiratory failure” – H2  “Trying to avoid descriptors that are a clinical decision, as opposed to a patient's state. The problem, of course, of measuring oxygen is if you're not going to be providing oxygen, you're not going to be measuring the oxygen, so that I think it's going to be even less likely that you'll have some measure of oxygenation.” – H1  “Outside the ICU, often non ventilated patients, this might be okay, we've got better measures in ventilated patients there.” – H3  “With the use of noninvasive ventilation or high flow, it's important that we also capture how much oxygen they're getting from that.” – H3 |
| **Multiorgan failure (SOFA/MODS score)** |  |
| **Advantages** |  |
| - **Routinely used in research and practice:** The SOFA was routinely used, particularly in practice and research in ICU settings. Therefore, there was high familiarity with the use and reporting of SOFA. It was considered to be the primary measure for multiorgan failure in critical care and a good measure for clinical evolution. Participants also noted that the measure had been used in trials in infections and pneumonia. - **Validated:** The SOFA score was developed and validated in the ICU, but could also be used in some patient populations outside of the ICU, including in the emergency department. The measure included a scoring system of organ function as well as organ support (e.g. vasopressors and ventilation). - **Ease of data collection:** The data from SOFA scores could be readily obtained from electronic records, and were easy to calculate. | “We always use only SOFA now. For 10 years we moved to SOFA and we only calculate SOFA and report SOFA both clinically and in the publications.” – H3  “We are more familiar with the SOFA.” – H3  “It is highly validated in the literature, most of literature in critical care are moving to SOFA as the primary way to measure the patient's multiorgan failure. Most of the physicians have already dealt with it.” – H3  “The SOFA was validated in use in the new sepsis definition and also is very useful for if you want to see the clinical evolution along the time. To calculate the Delta SOFA, there are much more data on using this index than probably the MODS.” – H3  “All the randomised clinical trials regarding infections, trials for an antibiotic or associated community acquired pneumonia and ventilator associated pneumonia use SOFA. And SOFA delta, SOFA changes as well.” – H3  “it's important that we use a commonly recognized measure, but we also need to accept that there are some limitations to it.” – H3  “It's easier to calculate than other scores that were available before. You can get it from the electronical medical records very quickly.” – H3  “it is part of the Sepsis-3 definitions and therefore I think it's core really to what we do in intensive care.” – H3  “I vote for SOFA” – H3  “Comparing to MOD score, the SOFA integrates also other things like ventilation and also this is important in a why comparing to the MOD score.” – H3  “The SOFA coincided with the classification proposed by the Berlin definition, in mild, moderate and severe.” – H3  “People seem to have more comfort and familiarity with SOFA.” – H3  “We've done this in several trials where we've used SOFA and particularly Delta SOFA to indicate deterioration.” – H3  “It would be advisable given the time constraints to use something that's already been validated.” – H3  “With Sepsis-3 definitions, some of the data from that did come from patient populations outside of the ICU. While SOFA was developed for patients in ICU. It has been used outside of ICU.” – H3  Some of these measures (like SOFA or ARDS classification) they are present in several scores; example P/F ratio is present in the SOFA, MODS, ARDS; so if you collect the different components of the score and not only the final score we can have all the data there. SOFA score is much more commonly used and in some places, it is collected daily for the assessment of organ failure.” – H2 |
| **Limitations** |  |
| - **Measurement errors related to clinical outcomes:** Death as a competing event would bias the SOFA score toward the better, while underlying comorbidities would bias the SOFA score toward the worse. The SOFA score would also be relatively insensitive for respiratory failure. While the SOFA score included some type of support for organ failure (i.e. ventilation, vasopressors), it did not include ECMO or kidney replacement therapy. Also, some organ systems were not assessed (e.g. gastrointestinal). - **Inadequate assessment of coagulopathy:** Coagulopathy was not adequately addressed with the SOFA score because the platelet count was less relevant in patients with COVID-19. Participants suggested that D-Dimer would be preferable to use. - **Limited use and validation in non-ICU settings:** Some participants suggested that the score may be of limited use and not well validated in settings outside of ICU. | “These scales are both 25 years old.” – H2  “What's not in it, is that coagulopathy occurs, the D-dimer levels.” – H2  “If we're looking at respiratory component, which is a major component of what happens to our patients. It's relatively insensitive. You've only got four points on there. So you're going to have high SOFA scores in patients who develop genuine multiorgan failure. But a lot of our patients are really single organ failure, they're respiratory failure. That's what really kills them on the whole.” – H3  “You can score SOFA at multiple time points. You can look at a temporal trajectory of it, but we also need to have some way of dealing with death. So, if you did something like total SOFA score or peak SOFA score and so forth, if somebody dies before they achieved that they may look unusually healthy on a SOFA scale.” – H3  “I'm just looking at my dad's background cause he was intubated and they classified him as having multiorgan failure, but he's already got a background of polycystic kidney disease and polycystic liver. Having those scores, his baseline is actually already starting in the middle. If anything, for whatever reason, his kidney function baseline has actually improved after he's come out of ICU.” – C3  “These measurements or these scores are validated for ICU patients. For patients outside the ICU it could be a problem. Maybe not all patients have a blood gas taken. So maybe we can use the peripheral saturation.” – H3  “Need to confirm this organ failure after 24 hours of mechanical ventilation with a specific level of expiratory pressure. This is a problem that is not considered in both networks, SOFA or MODS, except if you want to use the Delta SOFA during the first 24 hours.”  “You should attempt to go back into the patient's notes and work out what their baseline renal function was, what their baseline hepatic function was, and then almost subtract that from your score so that you are dealing with acute rather than chronic change.” – H3  “The duration of mechanical ventilation, or of vasopressor support or kidney replacement therapy) is not a good outcome because that die early can biased the result.”- H2 |

**Supplemental Digital Content 2: COVID-19-COS core outcome measure for shortness of breath: workshop presentation outline with review of measures, workshop themes and quotations**

WORKSHOP PRESENTATION OUTLINE

**BRIEF OUTLINE OF RATIONALE**

Shortness of breath was identified as a high priority outcome domain by patients, family members, health professionals and members of the general public through the international COVID-19-COS survey and consensus workshops.

**AIM OF THE WORKSHOP**

To decide on a core outcome measure for shortness of breath for trials in people with confirmed or suspected COVID-19

- Measure shortness of breath in a **meaningful**, **consistent** and **accurate** way
- Guide the development and evaluation of interventions
- Inform decision-making about treatment plans and patient care

**MEASUREMENT PROPOERTIES AND FEASIBILITY**

| **Property** | **Definition / example** |
| --- | --- |
| **Measurement** |  |
| Validity | Do the questions capture relevant experience of shortness of breath accurately? |
| Reliability | Are the scores consistent when measured under similar circumstances? |
| Responsiveness | Do the scores demonstrate change? |
| **Feasibility** |  |
| Patients can understand it | “I feel short of breath” versus “I have dyspnoea” |
| Easy to interpret the results | “reduction in shortness of breath” |
| Easy to administer | Electronic questionnaire / hardcopy questionnaire |
| Time to complete | Number of items, length of questions 1-3 questions |
| Clinicians understand it | “X patients are experiencing X level of shortness of breath” |
| Cost | Free / licence fee (Free) |
| Equipment | iPad versus paper |
| Available in different settings | Hospital and community, low/middle/high income countries |
| Easy to assess the results | Score calculated to combine all dimensions |

Adapted from the COSMIN-COMET Framework for selecting core outcome measures^26 35^.

**DEFINITIONS**

“A subjective experience of breathing discomfort that consists of qualitatively distinct sensations that vary in intensity …derives from interactions among multiple physiological, psychological, social, and environmental factors, and may induce secondary physiological and behavioral responses… dyspnea per se can only be perceived by the person experiencing it.” (American Thoracic Society 2012)

“A subjective symptom reported by patients. It is always a sensation expressed by the patient and should not be confused with rapid breathing (tachypnea), excessive breathing (hyperpnea), or hyperventilation. Dyspnea is most frequently described as shortness of breath, inability to take a deep breath, or chest tightness.” (Bass 1990)

**EXISTING MEASURES**

**Core outcome sets**

Source: COMET database ([www.cometinitiative.org](http://www.cometinitiative.org))

- None recommended

**Trials in COVID-19**

Source: Review of 301 published and registered trials in patients with COVID-19 (24 reported shortness of breath and of these two trials specified the measure used)

- PROMIS Dyspnoea functional limitations / dyspnoea severity (1 trial) at day 10
- Modified MRC (MMRC) scale (1 trial) at Day 7, 14, 28, 90

**Other common or endorsed patient-reported outcome measures**

Source: Literature search of systematic reviews and trials

- Baseline Dyspnea Index / Transition Dyspnea Index (BDI/TDI)
- Borg Dyspnoea Scale
- Borg Rating of Perceived Exertion
- Chronic Respiratory Questionnaire – Dyspnea subscale (CRQ-D)
- Clinical COPD CCQ
- Dypsnoea-12
- London Chest Activity of Daily Living Scale (LCADL)
- Multidimension Dyspnoea Profile MDP
- Pulmonary Function Status Dyspnea Questionnaire (PFSDQ-M)
- Shortness of breath questionnaire (SOBQ)
- St George Respiratory Questionnaire (SGRQ)
- Visual analog scale (VAS) dyspnea score

**Summary of measures**

The measures indicated in red/pink align best with the key items from the COSMIN-COMET framework

| **Measure** | **Items** | **Recall** | **Severity** | **Frequency** | **Function** | **Mental/emotion** | **Sensory** |
| --- | --- | --- | --- | --- | --- | --- | --- |
| **PROMIS-DFL** | 33 | 7 days | ♦︎ |  | ♦︎ |  |  |
| **PROMIS-DS** | 10,33 | 7 days | ♦︎ |  |  |  |  |
| **MRC / MMRC** | 1 |  | ♦︎ |  | ♦︎ |  |  |
| **BDI/TDI** | 24 |  | ♦︎ |  | ♦︎ |  |  |
| **Borg** | 1 | Current | ♦ |  |  |  |  |
| **CRQ-D** | (4) 20 |  | ♦ |  | ♦ |  |  |
| **CCQ** | 10 | 7 days | ♦︎ | ♦︎ | ♦︎ | ♦︎ |  |
| **Dyspnoea-12** | 12 | These days | ♦︎ |  |  | ♦︎ | ♦︎ |
| **LCADL** | 15 | Few days | ♦︎ |  | ♦︎ |  |  |
| **MDP** | 12 | Current | ♦︎ |  |  | ♦︎ | ♦︎ |
| **PFSDQ-M** | 40 | - | ♦︎ | ♦︎ | ♦︎ |  |  |
| **SGRQ** | 50 | 12 months/now | ♦ | ♦ | ♦ | ♦ | ♦ |
| **SOBQ** | 24 | - | ♦ |  | ♦ | ♦ |  |
| **VAS** | 1,2 | Current | ♦ |  |  |  |  |

| **Measure** | **Domains** | **Items** | **Scale** | **Recall** | **Cost** | **Validation** | **Considerations and issues** | |
| --- | --- | --- | --- | --- | --- | --- | --- | --- |
| **MMRC** | Rates shortness of breath according to different levels of activity | 1 | 5 point | NS | Free | Y | Simple to administer, correlates with other scales and scores of health status, useful at baseline  Grades are quite broad, lack of clear limits between grades, may be more difficult to detect small but important changes with interventions | |
| **MBorg** | Breathlessness during a particular task | 1 | 10 point | Current | License | Y | Correlation with lung function, sensitive to treatment effect, adjectives to determine level of breathlessness  Cost, primarily used in rehabilitation (during exercise), does not capture exercise/exertion explicitly Feasible? | |
| **VAS** | Amount of shortness of breath | 1 | Continuous | Current | Free | Y | Simple to administer, economical, greater precision.  Abstract (converting experience to a point on a line), subjectivity, less able to compare among individuals; burden – measure distance, primarily used in rehabilitation, does not capture exercise/exertion explicitly Feasible? |  |

NS, not specified; Y, yes; MMRC, Modified Medical Research Council Scale; MBorg, Modified Borg Scale; VAS, visual analogue scale

**The modified Medical Research Council Scale for dyspnoea**

1. I only get breathless with strenuous exercise
2. I get short of breath when hurrying on the level or walking up a slight hill
3. I walk slower than people of the same age on the level because of breathlessness or have to stop for breath when walking at my own pace on the level
4. I stop for breath after walking about 100 yards or after a few minutes on the level
5. I am too breathless to leave the house or I am breathless when dressing

Dennis E. Doherty, MD, FCCP, Mark H. Belfer, DO, FAAFP, Stephen A. Brunton, MD Leonard Fromer, MD, Charlene M. Morris, MPAS, PA-C, Thomas C. Snader, PharmD, CGP, FASCP. [Chronic Obstructive Pulmonary Disease: Consensus Recommendations for Early Diagnosis and Treatment](http://www.jfponline.com/uploadedFiles/Journal_Site_Files/Journal_of_Family_Practice/supplement_archive/JFPSupp_COPD_1106.pdf). Journal of Family Practice, November, 2006.

**DISCUSSION QUESTIONS**

1. Should the MMRC be recommended as the *core* measure for shortness of breath (compared with modified Borg and VAS)?

- Is it relevant to the patient’s experience?
- What aspects of shortness of breath are important/unique to COVID-19?
- Is severity and/or frequency important?
- Should exertion/exercise tolerance be captured?
- Can the specific activities (walking, dressing) be interpreted by the respondent?
- Is impact on emotional/mental health important?

1. What should the recall period be? (e.g. current, past week)
2. What should the metric be? (frequency, what time points, change over time, end of treatment)

WORKSHOP THEMES AND QUOTATIONS

| **Summary** | **Quotations** |
| --- | --- |
| **Capturing the dynamic nature of COVID-19** | |
| **Recognising the remitting trajectory of shortness of breath** | |
| Participants emphasised that shortness of breath in COVID-19 was unpredictable in terms of its onset and course. Patients experienced severe fluctuations in shortness of breath throughout the day, which “may be related to exercise, but not only related to exercise”. Overall, patients explained that they did not achieve normal functional capacity whilst they were experiencing shortness of breath. To best capture this experience, participants suggested the measure for shortness of breath to be administered at the same time of the day with a recall period of no more than 24 hours. | “Sometimes a few days you’d feel better, and then you’d feel worse again so it wasn’t consistent” – P1  “Within a day, even, the shortness of breath might change” – H1  “Even within a person, there was variability in terms of the symptoms…which seem to be part of the condition” – H1  “This is probably where I’m still struggling, eight weeks later… I’m not myself” – P3  “COVID-19 as all of us know, it’s a very dynamic condition” – H2  “She just doesn’t remember the last time she tried to walk upstairs. And so it’s not… and when she did last tried to do it, she didn’t have [dyspnoea]… she wasn’t in the same clinical condition that she is now. She’s in a different clinical condition… whereas with chronic disease, no, it doesn’t change very much from day to day” – H4  “It’s really variable… some days aren’t super bad, I might go the whole day think, I’m having a good day and then all of a sudden it starts again” – P4 |
| **Reflecting the debilitating severity** | |
| Participants suggested the need to distinguish shortness of breath in the acute stages of COVID-19 (up to about 12 weeks post-diagnosis) from the stable or recovery phase of COVID-19. In the acute stage, shortness of breath can be so debilitating that patients had no mental or physical capacity to do anything but focus on their breathing even at rest. One patient had to re-position the bed to be close to the bathroom and kitchen to minimise the steps required for the necessary daily activities. Other patients reported having to “schedule when to swallow because it interferes with breathing”. | “She feels like she has to schedule when she’s going to swallow because it interferes with her breathing, things like that… it’s difficult to imagine” – C2  “I set up a bed downstairs in the living room because it was right next to the bathroom right there” – P2  “I feel like I’m a hundred years old” – P3  “I could barely walk around the house” – P3  “People are experiencing breathlessness as a sensation, not necessarily, it may be related to exercise but not only related to exercise” – H4  “Just feeling kind of okay, but then I did this walk and it just killed me and I wouldn’t have predicted it or wouldn’t have gone if I knew it would have made me feel that bad” – P4 |
| **Defining improvement in terms of resumption of normal activities** | |
| Participants agreed that improvement in patients’ shortness of breath should be captured in terms of their ability to resume usual activities, and should be included in the descriptors to grade the severity of shortness of breath. They also noted that the activities assessed in the measure should adequately reflect the limitations of activity they could do in the context of COVID-19. For example, assessing shortness of breath while walking up a hill (which was a descriptor in the MMRC) for a patient in the acute phase of COVID-19 was deemed irrelevant, as they “would not even be able to imagine it at that point”. Also, some suggested to remove “leaving the house” from the MMRC descriptor because of the need for self-quarantine. | “you’re sick and bedridden so you can’t think and go, ‘how would I feel when I’m walking up a hill?’” – P2  “I’m coughing, I’m feeling very fatigued and run down with muscle aches and pains. And it just seems like you wouldn’t be leaving the bed and testing out your shortness of breath” – P2  “Strenuous exercise is a distant dream at the moment” – P4  “I’ve been in the house pretty much for seven weeks and I don’t think to leave the house for four, five weeks maybe. Walking up a hill… I don’t even know. It’s difficult to say” – P4  “Thinking about the course of the condition… number four on the scale [MMRC] isn’t granular enough, and maybe something around activities of daily living, albeit that you can’t go out to work or college or whatever, but nevertheless, some of those scales have more granularity in terms of dressing, in terms of getting upstairs, cooking” – H2 |
| **Comprehending the full experience of shortness of breath** | |
| **Gaining awareness of an unfamiliar symptom** | |
| Patients recalled difficulty in describing the feeling of shortness of breath as they had no experience of such sensation. One patient “only learnt that shortness of breath was a symptom that [they were] having in week two” and had previously been “telling the nurse in other roundabout ways that [they were] struggling with [their] lungs”. Even for those who had an underlying respiratory condition such as asthma, they found that the experience of shortness of breath because of COVID-19 was “different than what [their] normal short of breath is”. | “I only learnt that shortness of breath was a symptom that I was having in week 2 when I was constantly telling the nurse in other roundabout ways that I was struggling with my lungs or my breathing or everyday things. It was only when in week two, the nurse was like ‘you mean shortness of breath?’ and I was like, oh, I didn’t realize that was what it was like, because I’ve never had that symptom before…so if someone on day one gave me this [questionnaire] I’d be like I’m fine” – P2  “Okay I’m feeling tired, but this one is different” – H2  “It is so different than what her normal short of breath is” – C2 |
| **Acknowledging the different sensations of dyspnoea** | |
| Across the groups, patients reported varying sensations of shortness of breath. Some described it as “needing more air” and “tightness of the chest”, while others felt like “being smothered” and “just exhausted”. | “If the patient have asthma, at that moment they can say ‘okay this is a different shortness of breath than the normal’” – H2  “I was conscious of the lung” – P4  “just exhausted is what it sounds like” – H4  “I’m feeling chest tightness” – P4  **“**Patients reported the sensation of breathlessness in different ways like smothering sensation or a tightness sensation or a constricted sensation” – H4 |
| **Addressing the impact on mental health** | |
| Participants were concerned about the impact of COVID-19 related shortness of breath on the mental health of patients. More specifically, they noted the vicious cycle between shortness of breath due to impaired respiratory function and anxiety, as one exacerbates the other. Patients reported being turned away at the emergency department as “just anxious” despite having severe dyspnoea and felt dismissed for not having “a real symptom”. | “She feels like her shortness of breath interferes or impacts…her mental state, she gets really foggy and has a memory issue because she can’t get enough oxygen in and because it goes for such a long period of time” – C2  “It’s [mental component of dyspnoea] probably the most difficult symptom that she has on a everyday basis because it affects everything that she does” – C2  “I’m not anxious, I’m sick” – P3  “It’s something that survivors find very frustrating and the doctors are like ‘oh, this must be anxiety’” – P3  “Anxiety is a real symptom” – H4  “They just said, ‘well it’s normal to be anxious’” – P3 |
| **Ensuring ease of implementation** | |
| **Minimising burden on patients with a simple and short measure** | |
| Identifying a simple and short measure was needed to facilitate administration and comprehension of the question: “something very simple that the patients can answer very quickly and the people who are collecting the information can collect and process it”. Participants agreed that the MMRC was simple to administer. | “Mission here is to find a simple to use across the world scale that is tried and tested” – H1  “easiest and simplest to complete” – P1  “you need something very simple that patients can answer very quickly and the people who are collecting the information can collect that information and… process it” – P3  “If… you needed someone to update every day with a response, it needs to be quite quick and easy to do” – P1  “There are other tools that are more comprehensive, and they are 50, 60 questions. When you are trying to collect that data, they are impossible to fill” – H3 |
| **Allowing flexibility in time points based on the population and intervention** | |
| Rather than having a set frequency and timing of administration, participants felt that it should be determined by the research team in discussion with patients. This may require consideration of the trajectory of shortness of breath as reported by the trial population (for example, in the acute phase it may change more rapidly compared with the longer term or recover phase). Considerations related to the intervention, expected treatment effect, and the severity of COVID-19 should be considered to capture meaningful patient-reported data on shortness of breath. | “It is really up to what these study teams and what their study protocol decide” – H1  “Really important to justify the time points” – H3 |
| **Requiring validation** | |
| Due to the sudden onset and rapid progression of the pandemic, participants acknowledged there were no measures specific to COVID-19. Regardless, validity of a measure was seen as a crucial element in establishing a core outcome measure. While MMRC may be the best available option among the existing measures for dyspnoea, some suggested minor changes to improve its face and content validity for use in COVID-19. Participants also acknowledged that validation would need to be an iterative process, where accumulation of data through use in clinical trials will progressively build evidence to support the validity of a measure, particularly in the context of the global pandemic where there was limited time to develop and validate a new measure prior to use. | “It isn’t perfect, but I suspect it’s probably the best we have” – H1 |

P, patient, H, health professional; C, caregiver; number refers to the breakout group ID

**Supplemental Digital Content 3: COVID-19-COS core outcome measure for recovery: workshop presentation outline with review of measures, workshop themes and quotations**

WORKSHOP PRESENTATION OUTLINE

**BRIEF OUTLINE OF RATIONALE**

Recovery was identified as a high priority outcome domain by patients, family members, health professionals and members of the general public through the international COVID-19-COS survey and consensus workshops.

**AIM OF THE WORKSHOP**

To decide on a core outcome measure for recovery for trials in people with confirmed or suspected COVID-19

- Measure recovery in a **meaningful**, **consistent** and **accurate** way
- Guide the development and evaluation of interventions
- Inform decision-making about treatment plans and patient care

**MEASUREMENT PROPERTIES AND FEASIBILITY**

| **Property** | **Definition / example** |
| --- | --- |
| **Measurement** |  |
| Validity | Do the questions capture relevant experience of recovery accurately? |
| Reliability | Are the scores consistent when measured under similar circumstances? |
| Responsiveness | Do the scores demonstrate change? |
| **Feasibility** |  |
| Patients can understand it | “I have mostly recovered” versus “I have recovered 75%” |
| Easy to interpret the results | “increase in self-reported recovery” |
| Easy to administer | Electronic questionnaire / hardcopy questionnaire |
| Time to complete | Number of items, length of questions (1-3 questions) |
| Clinicians understand it | “X patients are experiencing X level of self-reported recovery” |
| Cost | Free / licence fee (Free) |
| Equipment | iPad versus paper |
| Available in different settings | Hospital and community, low/middle/high income countries |
| Easy to assess the results | Score calculated to combine all dimensions |

Adapted from the COSMIN-COMET Framework for selecting core outcome measures^26 35^.

**DEFINITIONS**

“How long it takes to recover i.e. feeling better, no longer having symptoms” (COVID-19-COS International Survey)^24^

“Feeling better and having no symptoms, doing usual activities and have returned to their usual state of health prior to COVID-19 illness” (COVID-19-COS International Consensus Workshops)^19^

“The act of regaining or returning toward a normal or healthy state” (Merriam-Webster Medical Dictionary <https://www.merriam-webster.com/dictionary/recovery>)

**EXISTING MEASURES**

**Core outcome sets**

Source: COMET database ([www.cometinitiative.org](http://www.cometinitiative.org))

- Survivorship in Acute Respiratory Failure (Needham et al., 2017)^37 38^: separate core outcome measures recommended for each of 8 domains (survival, HRQoL, mental health, pain, cognition, physical function, muscle/nerve function, pulmonary function)

**Trials in COVID-19**

Source: Review of 301 published and registered trials in patients with COVID-19 (76 reported recovery and of these one trial used a patient-reported outcome measure

- ”Days to self-reported recovery” – phone interview at day 30 including daily life limitations (no further information provided)

**Other common or endorsed patient-reported outcome measures**

Source: Literature search of systematic reviews and trials

- Brief Illness Perception Questionnaire (BIPQ)
- Clinical COPD Questionnaire (CCQ)
- COPD Assessment Test (CAT)
- EuroQol-5D (EQ-5D 3L or 5L)*
- FRAIL Scale
- Functional Performance Inventory (FPI)
- Hospital Anxiety and Depression Scale (HADS)*
- Impact of Events Scale – Revised (IES-R)*
- International Physical Activity Questionnaire (IPAQ)
- London Chest Activities of Daily Living Scale (LCADL)
- Manchester Respiratory Activities of Daily Living Scale (MRADL)
- Maugeri Respiratory Failure Questionnaire (MRF-28)
- Nottingham Health Profile (NHP)
- Patient Health Questionnaire (PHQ-9)
- Perceived Quality of Life (PQOL)
- Physical Activity Scale (PAS)
- Physical Activity Scale for the Elderly (PASE)
- Pulmonary Functional Status and Dyspnea Questionnaire (PFSDQ)
- Pulmonary Functional Status Scale (PFSS)
- Quality of Life Scale (CASP-19)
- Reintegration to Normal Living Index (RNLI)
- Seattle Obstructive Lung Questionnaire (SOLQ)
- Short Form-12 (SF-12)
- Short Form-36 v2 (SF-36)*
- Sickness Impact Profile (SIP)
- St George’s Respiratory Questionnaire (SGRQ)
- Symptom Checklist-90-R (SCL-90-R)
- WHO Disability Assessment Schedule 2.0 (WHODAS 2.0)

*measure recommended by Needham 2017 for their respective domain

**Summary of measures**

The most frequently reported measures were included in the comparison table.


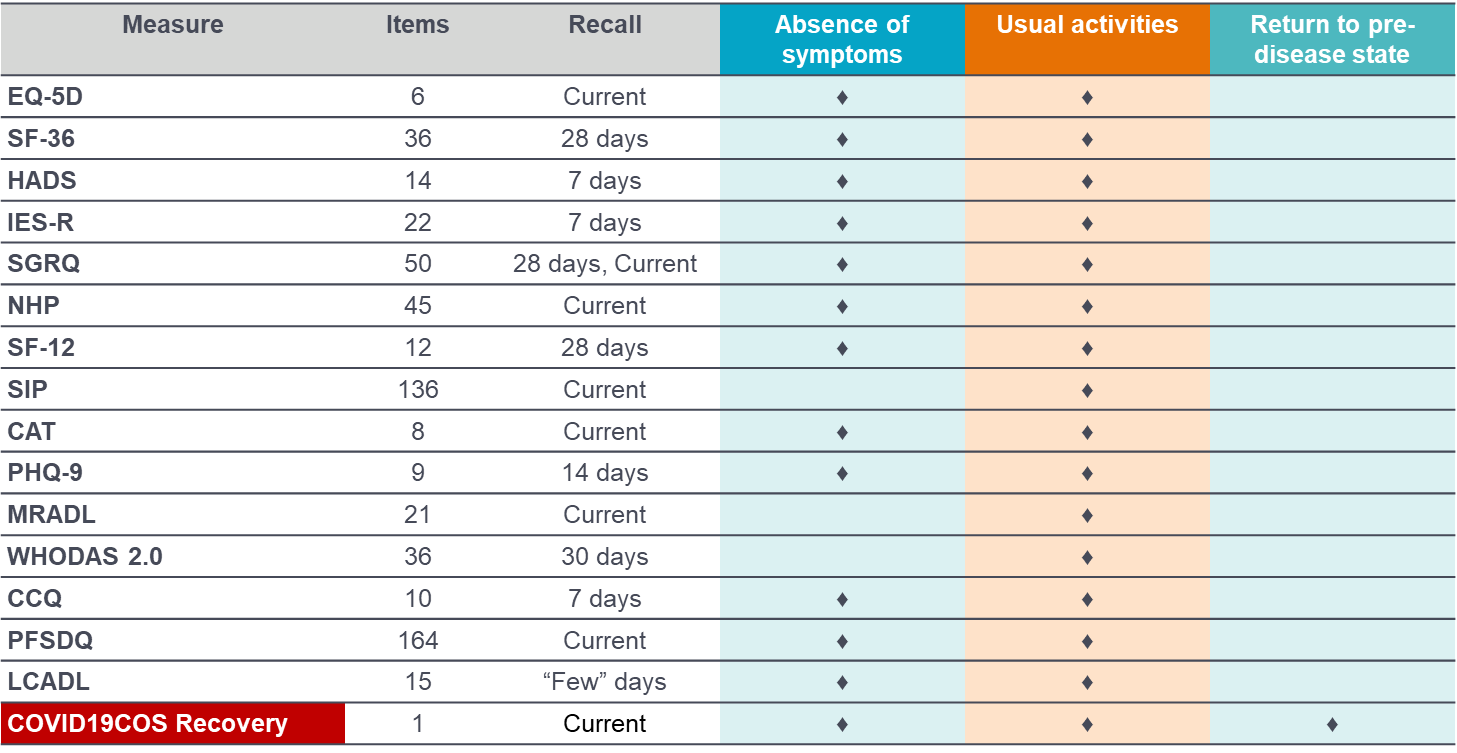


**COVID-19-COS RECOVERY (PROPOSED MEASURE PRESENTED AT THE WORKSHOP)**

Have you completely recovered from COVID-19?

**Complete recovery means you are feeling completely better, are having no COVID-19-related symptoms, are doing your usual activities and have returned to your usual state of health (prior to your COVID-19 illness).*

Please choose the answer that best describes you:

| Not recovered at all | Recovered a little | About half recovered | Mostly recovered | Completely recovered |
| --- | --- | --- | --- | --- |
| ◯ | ◯ | ◯ | ◯ | ◯ |

**DISCUSSION QUESTIONS**

1. Should COVID-19-COS Recovery be recommended as the *core* measure for recovery?

- Is it relevant to the patient’s experience?
- What aspects of recovery are important/unique to COVID-19?
- Is it easy to understand and complete?
- Is it applicable across the full spectrum of disease and across care settings?
- If “no”, what measure would you recommend and why?

1. What should the metric be? (what time points, frequency, change over time, end of treatment)

WORKSHOP THEMES AND QUOTATIONS

| **Summary** | **Quotations** |
| --- | --- |
| **Recognising the diverse meaning of recovery** | |
| **Being relevant and comprehensible to the patients’ own experience** | |
| Participants emphasised that recovery should be measured in a way that is “meaningful” and “captures the essence” of the patient experience, including those who were hospitalised or were managed at home or in primary care settings. Participants indicated that the proposed measure was “very relevant to the patient experience,” “encompassed the absence of symptoms, and return to normal physiologic activity,” and “easy to understand.” It was suggested that the term ‘recovered’ may be too “clinical” and “feeling better” may be better understood by patients; however, this term was considered “too general.” One patient expressed ‘COVID-19 related symptoms’ sounded “very medical.” The response scale of the proposed recovery measure (Supplementary File) was regarded as easy to complete, though some patients did not “get a good sense of the differences between categories” and found ‘about half recovered’ unclear. Some advised to change the question to “how much have you recovered” to capture the “degree of recovery.” | “I think sometimes with some clinicians, hospital-based recovery means, this patient no longer needs my care and they've gone away and I don't see them again. But what that means in terms of that patient's experience at home or with primary care just doesn't come into it.”- P3  “Off the top of my head right now and thinking about my recovery experience, I think that the core measure is a good one. I can't think of any alternatives.” – P1  “Some of the things that came up was firstly that we thought the question was really relevant to the patient experience.”- H4  “I think the questionnaire makes a lot of conceptual sense. If someone asks me those questions, I would be able to answer. They're not hard.” – P3  “Also, around the use of terminology, I think there was some comments that perhaps recovered is quite a clinical term and something such as are you better might be more patient centered.” – H4  “The word ‘better’ is too general of a term. There needs to be some commonplace between recovery and better.” - P2  “I think you need to think about whether you want to measure the degree of recovery and change the question in terms of how much have you recovered. And then you could think of not recovered a little bit, a lot, et cetera, or the answer would be yes or no. About half recovered, that is a weird concept. You cannot be half recovered, you're recovered or not. That just doesn't exist.” – H4 |
| **Allowing interpretation within individual contexts** | |
| For the measure to be relevant to all patients, participants recognised that “[COVID-19] means different things to different people.” COVID-19 could have a “profound effect” on a patient’s “comorbidities” and recovery would need to be considered in terms of their “pre-disease state” of health before being diagnosed with COVID-19. There were concerns that ‘returned to your usual state of health’ did not reflect patients’ “complex and fluctuating” health and ‘usual’ was recommended to replace “previous”. Participants agreed the measure should remain “open-ended” and advised against providing examples of health status or symptoms because of the “very heterogenous” experiences of patients. Similarly, while providing examples of ‘usual activities’ could “help give context”, it was considered “really hard to nail a good example” that was “meaningful”, applicable to all patients and still allowed “some degree of subjectivity”. | “I would look at that and I would want to qualify my answer in relation to underlying diseases. I might be better from Covid, but if it's had a profound effect on my other underlying diseases then how do I answer that?” – P5  “One should in fact consider as one of the outcome measures not necessarily a return to usual state of health, but the pre-disease baseline state of health, which in its own right may fluctuate.” – H5  “I would have to say that I kind of agree that it has to be more of a broader spectrum in terms of not everyone experiences the same symptoms.” – P2  “Given that this is a personal experience and given the patients have very different background, they’re in different health states when they get COVID, it’s really hard to nail a good example, because if you think about exercising, for example, well, some people could not exercise before the disease. Yeah, I understand the concern, but I’m not sure how we can give good examples” – H2 |
| **Reflecting a return to normality** | |
| Participants conceptualised recovery as the return to “whatever life you had pre-COVID.” They suggested that the measure should not only determine if patients were “symptom free” but should assess the extent to which patients could participate in their normal activities, such as exercise or usual employment. Some patients who had been discharged from hospital felt that “even though I was mostly physically recovered, I just can't perform at my job the way I used to.” | “When we talk about recovery, when we talk about being symptom free, especially the use of the words, doing your usual activities, I found that I can't really do my usual activities the same as I could before, because of some of the damage that's happened to my body post-virus in recovery.”- P2  “I think it's really ... I don't want to say sensitive but I think it's really thoughtful to say that you are mostly recovered or complete recovered, meaning that you've returned to your life, whatever life you had pre-Covid, is a big deal for a lot of people.” – C5  “I think it encompasses the elements that need to be addressed, in other words, absence of symptoms, return to normal physiologic activity which has already been alluded to, so relevant. And the pre-disease state addressed as well, which is very relevant because depending on where one is in the world, there are many people who may in fact have various comorbidities.” – H5 |
| **Incorporating physical and mental wellbeing** | |
| Participants advocated for including the “cognitive and psychological aspect of recovering” into the measure since a patient could be “physically fine” but be “psychologically impaired” or struggle with “analytical thinking and lateral thinking”. They believed patients “may default to thinking of physical health rather than psychological health” when thinking about their recovery, and that the proposed measure should explicitly instruct them to consider their “state of health and state of mind”. | “The point about cognitive wellbeing, I think is a good one. For me personally, my job requires analytical thinking and lateral thinking.”- P1  “I wondered if the term usual state of health could be refined to say something like usual state of physical and psychological health. Of course, I know that those words may not be understandable by a patient, but somehow putting in some sort of adjective or descriptor to help them understand that we mean their entire state of health, not just defaulting to physical.” – H1  “We also know that at least survivors of critical illness don't have clear insights into their cognitive impairments when we do performance based testing versus patient reported outcomes. I know that we specifically want a patient reported outcome. So this may be a little insensitive to patients appreciating that they have cognitive deficits.” – H1 |
| **Addressing dynamic trajectories of recovery** | |
| **Capturing the ‘wax and wane’ of symptoms** | |
| Whilst recovering, some patients experienced “fluctuating” symptoms that could “change within hours”. Health professionals remarked “we assume that in most conditions, the road to recovery is a steady slope upwards. Whereas this is up and down and back up again.” Some questioned if the proposed measure could account for relapses or fluctuating symptoms, which was clarified by others who explained that the researchers may “average the data from a group of participants,” administer the measure multiple times, or embed a specific recall period such as the past week to “overcome day to day variations”. Participants suggested patients could also keep a daily “diary” or respond as “compared to the last time you reported this.” | “I think it's important to have five options because my experience of recovery and on week eight, there's been relapses. So there have been times when I thought I've been recovered and in fact, another symptom comes back.” – P2  “We spoke about the issue of relapse. I was asking myself, "Okay, how is this question really capturing it?" Because it does have a grading in your response, but there still remain a lot of subjectivity. How do you rate it when you have the relapse? Do you say, recovered a little or about to recover?” – H2  “It's about how you feel now. That's what we try to measure. And some patients will have a good day and they will say, I feel recovered. And some patients will have a bad day and they will say, no, I don't feel recovered. If we're talking about clinical trials where in the end we average the data from a group of patients.” – H4  “A concept that's coming up of timeframe that you've probably been trying to avoid, but this is not a smooth recovery. So are you asking people, how do you feel today? How do you feel this week? How do you feel right this second? I think that's a fabulous concept that somehow needs to get factored into this. It obviously also depends on where you're going to be using this question. Is it just once or are you using it to query patients on a diary or device?” – H4 |
| **Distinguishing other contributing factors to recovery** | |
| There was a need to distinguish patients’ recovery from their other comorbidities or “indirect effects of the pandemic” to ensure an assessment of recovery from COVID-19. Other illnesses such as cancer “probably affect” their symptoms. Participants noted the phrase ‘recovered from COVID-19’ could be interpreted by patients as “not only the acute disease, but also the pandemic and its indirect effects, economic recession, mental burden, lockdown activities” and recommended clarifying the scope of the measure as recovery from the “COVID-19 illness”. | “I have cancer as well so I'm on other things which I think probably affects it.” – P2  “Because when you read, ‘Have you completely recovered from Covid-19?’ What does that mean to your own disease or your family condition or to other economic effects of disease or if you are talking about the specific getting ill and getting hospitalized and so on.” – H5 |
| **Assessing the long-term impact** | |
| Participants stated that recovery could often take longer than the “two weeks” that was typically indicated by health professionals or the news media. Elderly patients, those who had been hospitalised and even patients with mild cases could experience a “long road of physical rehabilitation of 2-3 months or more.” They advocated greater attention to long-term recovery in trials stating, “there’s quite a large minority of people who have not got there in two weeks... if you don’t measure [it], it doesn’t exist.” Some patients also questioned “whether there is a total recovery with [COVID-19]”. | “In the long-term, the multifaceted aspects of post intensive care syndrome, which can persist for many months or years after discharge, may also manifest in reduced exercise capacity, independence with activities of daily living, and health-related quality of life.”- H1  “We know a bit more about what happens during those two or three weeks, but we don't know what happens longer than that. And then if we have more of a severe case where these are patients who were intubated and end up having to do a lot of physical medicine rehab, do they actually recover fully or not?”- H2  “We don't know how a normal recovery looks like at the moment to be fair. I think a lot of us are not even sure whether there is a total recovery with this whole thing. And that's the scary part.”- P4 |
| **Ensuring feasible and universal implementation** | |
| **Having broad applicability to all care settings and populations** | |
| Recovery was relevant to all patients with COVID-19 and it was noted that “a lot of people don’t get hospitalised.” They considered it “better to use a single scale [for recovery] to be able to compare the different trials” across care settings and populations. The proposed measure was believed to be “very easy for low and middle-income countries or resource limited settings,” and was considered to be adequately “simple” to translate. Some advocated for a measure that would be “appropriate for paediatric use” and a measure that could be completed “by proxy” (i.e. by a caregiver or health professional) in cases where patients could not complete the measure on their own, for example if on a ventilator. | “We know, we believe that the scales used in the trials are valued, but it's better to use a single scale to be able to compare the difference trials and diverse populations.” – H1  “For younger children, is this designed to be administered by a parent or a carer or could it be? And I wondered about any kind of visual. I wondered about something like a circle, which is half completed, or three quarters completed or fully completed. I think translating this will be pretty simple, but a visual that is universal, irrespective of which country or which language it's administered in might have some advantage. – H3  “I think it is kind of setting even patients that come from what treated outpatient or inpatient, but maybe because this is such an objective way of measuring what each one is feeling.” – H2 |
| **Encompassing the full spectrum of disease** | |
| Given the different degrees of severity of COVID-19, participants emphasised “applicability across all types of severity” of COVID-19. Overall, participants felt the proposed measure would be “easy (to use) across the full spectrum of disease” but some were uncertain if it would “have the same value in positive COVID-19 patients with moderate-mild disease” or in asymptomatic cases as patients would need “to have some element of symptom enough for you to notice to be able to complete this.” It was also argued that all patients (including those with mild or moderate illness) would still be able to make comparisons to their previous baseline of health and patients indicated they could distinguish the difference between the response categories. | “I think it's a good way of measuring, even though those that were more critically healed, maybe they evaluate the limitations in a different way of comparing to those that did not have such a severe disease. But anyway, this is a subjective way of measuring the impact of the disease in each one.” – H2  “I think that I would have been able to tell the difference. I would say my case was sort of medium intensity, and there might've been like three or four days difference between feeling maybe 90% better to feeling a hundred percent better.”- P2 |
| **Minimising burden of administration** | |
| The measure had to be “succinct and simple for global use.” A “blunt tool measuring a complex construct” was reasonable because it would not be feasible nor relevant to “delve into all domains [of recovery] in every trial.” There were concerns that a composite measure may be “reductionist” and did not capture “the richness of the patient experience”. Some suggested “to break it out into the sub domains for greater specificity” and to account for patients recovering “in terms of different aspects at various times”. Some suggested to focus only on “usual activities” which could “be the final pathway affected by symptoms and return to previous health state.” Overall, minimising complexity and time for completion was expected to support broader implementation and higher response rates. It was suggested that triallists could choose to use other detailed measures for recovery, for example if recovery was a primary outcome. Some recommended to use a “fixed time point that can be measured consistently across groups for all trials” with “90 days after randomisation” specified by some; however, others noted this would not be feasible as it would depend on other considerations including the intervention and trial duration. | “The different components may not all recover at the same pace. And I think that is a very important point because one was looking effectively with those three elements as a composite end point. A return to usual activities is felt to probably be the most relevant element and as has already been alluded to, the physical and psychological elements need to be in fact considered.” – H5  “Sometimes it can be handy to break it out into the sub domains for greater specificity, to learn a little bit more about some of the nuance or try to tease out some of the nuances to what sort of outcomes patients are reporting.” – H3  “When I get one of my new patients into the trials is usually about 150 questions and imagine if you send that person a questionnaire home to fill out, multiple questions and they don't have interest or financial motivation to do it, the majority of patients will just not do it. People don't even fill out the census so just think about 140 questions.” – H5  “Just to sort of speak in favor of a simple blunt tool that lets you capture the temporality and where what it means is kind of in the eye of the beholder. Researchers may not love that as much because it lacks granularity but design a more granular study next time. If you, if you're interested in the question and now, you need to ask the question, ask the question in a focused way.” – H4  “I spend a lot of my day trying to convince clinical trialists and critical care to measure anything after hospital discharge, and I think there's lot of resistance. I would favor because this is the minimum core mandatory thing. People can measure much more often than they want, but I would favor a single time point at 90 days after randomization, not after hospital discharge, but after randomization. I think many clinical trialists are beginning to accept that they have to follow patients for at least 90 days. So I think that would work into workflow.” -H1 |
| **Considering potential use in other populations and contexts** | |
| Some believed the measure had potential to be used in other patient populations – “ideally I would like to see a tool that can then be applied to all relevant ICU trials that follow severe illness.” Some were uncertain about the use of a new measure as this would prevent comparison of COVID-19 to other diseases; however, it was suggested that other existing measures could be used to facilitate comparisons of related outcome domains such as quality of life. | “So obviously we're talking about COVID-19, but if we're talking about core outcomes in the more general sense, I'm not sure that you need the COVID-19 in the question… I guess, as a researcher, ideally I would like to see is a tool that can then be applied to all relevant ICU trials that follow severe illness trials.” – H1  “If we use something that's very different from what's used in other studies then there is a potential problem with comparability there. I think this measure is very useful, but I would advise perhaps having a subsidiary measure which would be applicable across every trial. The EQ5D seems like a reasonable measure, I would take other people's advice of which specific measures to go for, but something generalizable that has been widely used in the past would be helpful in addition to something like this.” – H1  “I'd be really, really keen to see some dissemination around the true recovery to people like government and also to GPs.” – P3 |

P, patient, H, health professional; C, caregiver; number refers to the breakout group ID

Based on the workshop discussion, the following changes were made to the new measure that was presented at the workshop (above). These are explained as follows:

- The recall period was specified to “today” for ease of response.
- The terms “COVID-19/COVID-19 illness” were omitted from the measure to have potentially broader relevance to other patient populations.
- The phrase “have you recovered from COVID-19” was changed to “how recovered are you from your illness” to ensure it matched the response scale and captured the degree of recovery, and to distinguish the recovery from COVID-19 illness from other indirect effects of the pandemic.
- In the stem, “COVID-19-related symptoms” was changed to “symptoms related to your illness” for ease of comprehension.
- In the stem, “usual activities” was changed to “usual daily activities” to give patients more context of the included activities in the absence of examples of activities.
- In the stem, “are doing your usual” was changed to “can do your usual” so that patients’ responses were not impacted by the need to self-quarantine which may prevent them from “doing” their usual activities.
- In the stem, “usual state of health” was changed to “previous state of health” to more easily allow patients to make comparisons to their previous health state and to acknowledge patients’ fluctuating states of health that may not be captured by the term “usual”.
- In the stem, “state of health” was broadened to “state of health and mind” to explicitly instruct patients to consider both their physical and mental recovery.
